# Discriminant canonical tool for inferring the effect of αS1, αS2, β, and κ casein haplotypes and haplogroups on zoometric/linear appraisal breeding values in Murciano-Granadina goats

**DOI:** 10.3389/fvets.2023.1138528

**Published:** 2023-07-07

**Authors:** Javier Fernández Álvarez, Francisco J. Navas González, José M. León Jurado, Antonio González Ariza, María A. Martínez Martínez, Carlos Iglesias Pastrana, María G. Pizarro Inostroza, Juan V. Delgado Bermejo

**Affiliations:** ^1^National Association of Caprine of Murciano-Granadina Breed (CAPRIGRAN), Granada, Spain; ^2^Department of Genetics, University of Córdoba, Córdoba, Spain; ^3^Agropecuary Provincial Centre, Diputación Provincial de Córdoba, Córdoba, Spain; ^4^Animal Breeding Consulting, S.L., Córdoba Science and Technology Park Rabanales, Córdoba, Spain

**Keywords:** data mining, SNPs, caprine, discriminant canonical analysis, zoometrics

## Abstract

Genomic tools have shown promising results in maximizing breeding outcomes, but their impact has not yet been explored. This study aimed to outline the effect of the individual haplotypes of each component of the casein complex (αS1, β, αS2, and κ-casein) on zoometric/linear appraisal breeding values. A discriminant canonical analysis was performed to study the relationship between the predicted breeding value for 17 zoometric/linear appraisal traits and the aforementioned casein gene haplotypic sequences. The analysis considered a total of 41,323 zoometric/linear appraisal records from 22,727 primiparous does, 17,111 multiparous does, and 1,485 bucks registered in the Murciano-Grandina goat breed herdbook. Results suggest that, although a lack of significant differences (*p* > 0.05) was reported across the predictive breeding values of zoometric/linear appraisal traits for αS1, αS2, and κ casein, significant differences were found for β casein (*p* < 0.05). The presence of β casein haplotypic sequences GAGACCCC, GGAACCCC, GGAACCTC, GGAATCTC, GGGACCCC, GGGATCTC, and GGGGCCCC, linked to differential combinations of increased quantities of higher quality milk in terms of its composition, may also be connected to increased zoometric/linear appraisal predicted breeding values. Selection must be performed carefully, given the fact that the consideration of apparently desirable animals that present the haplotypic sequence GGGATCCC in the β casein gene, due to their positive predicted breeding values for certain zoometric/linear appraisal traits such as rear insertion height, bone quality, anterior insertion, udder depth, rear legs side view, and rear legs rear view, may lead to an indirect selection against the other zoometric/linear appraisal traits and in turn lead to an inefficient selection toward an optimal dairy morphological type in Murciano-Granadina goats. Contrastingly, the consideration of animals presenting the GGAACCCC haplotypic sequence involves also considering animals that increase the genetic potential for all zoometric/linear appraisal traits, thus making them recommendable as breeding animals. The relevance of this study relies on the fact that the information derived from these analyses will enhance the selection of breeding individuals, in which a desirable dairy type is indirectly sought, through the haplotypic sequences in the β casein locus, which is not currently routinely considered in the Murciano-Granadina goat breeding program.

## 1. Introduction

Goat farming now extends to almost all countries worldwide due to the competitive prices and the high nutritional value of the products (especially milk) derived from this species, attracting new investment companies and farmers (1). Developing countries account for the largest fraction of the world goat census (over 90%) due to the great adaptability potential of the species to marginal territories, and its ability to thrive under adverse climatic conditions and within low-tech farming systems (2). Such a scenario contrasts with that of Europe and North American countries, where highly developed and intensive conditions rule the goat industry. This defines a highly focused milk production industry supported by the exploitation of high-yielding breeds genetically managed under the scope of breeding schemes (3). However, the development of genetics, nutrition, and animal management in the goat is rather limited compared with the level of integration and technification of these methods in other ruminant species (4).

Thus, the casein cluster is a genomic region of great interest in the goat, and has shown significant genetic diversity and differentiation among small ruminant populations (5). This genetic variability can be explained by the effect of several mutations on gene expression levels (6). In goat species, the casein complex comprises a series of genes located on chromosome 6. Specifically, casein genes are encoded by four loci (CSN1S1, CSN1S2, CSN2, and CSN3) clustered within the 250 kb segment of this chromosome (7). Casein single nucleotide polymorphisms (SNPs) act as genetic units that are closely linked through epistatic relationships (8). These markers are transmitted as haplotypes (9). The genetic polymorphism of the casein complex (αS1, β, αS2, and κ-casein genes), either in the form of SNPs, haplotypes, or haplogroups, associates with specific productive traits (milk yield, components, and lactation curve parameters) of interest from an economic and research point of view (10, 11).

The consideration of casein haplotypes rather than the use of a single gene or genetic marker has been suggested to maximize the comprehension of heritable mechanisms and how they affect the expression of functional traits related to milk yield, the production of its different components (protein, fat, dry extract, and/or lactose), cumulative milk production, and the greater or lesser presence of somatic cells (10, 12). Although SNP, haplotype, or haplogroup associations across casein genes and casein variants with milk production traits have been previously reported (10), the relationship of casein haplotype variants with morphometry and linear appraisal has not been investigated in depth.

In 1993, the American Dairy Goat Association introduced the Linear Appraisal System for dairy goats to improve production yields. However, the implementation of the Combined Goat Index and Morphological Index in the selective nucleus of the rustic Murciano-Granadina goat breeders’ association (CAPRIGRAN) did not occur until 2010 (13, 14). The breed’s morphology has evolved toward dairy type, making it necessary to develop a specific zoometric/linear appraisal scale to represent the breed population accurately. This led to the optimization and validation of the scale, enabling comprehensive genetic evaluations of hereditary components and correlations across zoometric linear appraisal traits (15, 16). When evaluating breeding does, four areas—structure and capacity, dairy conformation, mammary system, and legs aplomb—are scored and weighted at 25, 15, 40, and 20%, respectively. For breeding bucks and goats that have not given birth, only three areas—structure and capacity, dairy conformation, and legs aplomb—are evaluated, with relative weights of 50, 20, and 30%, respectively. The final score can range from 0 to 100 points, based on the relative scores obtained by the animal in each area.

The phenotypic relationship between zoometrics and dairy production (either milk yield, components, or even transformed products, such as cheese) has been investigated (17–19) and tools seeking the optimal dairy goat type have been developed (13, 15, 16, 20, 21). As a result, some methods have been developed for predicting daily milk production and the performance of milk components from morphometry and linear appraisal (22, 23). However, the role that dairy-linked genes, such as those in the casein complex, traditionally play in growth or zoometrics remains unexplored, especially in species other than cow.

In this context, the present study aims to develop a discriminant canonical analysis (DCA) tool that outlines the effect of the individual haplotypes of each component of the casein complex (αS1, β, αS2, and κ-casein) on zoometric/linear appraisal breeding values. The information derived from the present analyses will help to plan strategies that support the standardization and improvement of the productive capacity of this native goat breed to seek the consolidation of the breed in the international dairy goat panorama.

## 2. Materials and methods

### 2.1. Zoometric and linear appraisal breeding value prediction

#### 2.1.1. Pedigree matrix and linear appraisal records

The Murciano-Granadina whole-pedigree data file comprised 279,264 animals (266,793 does and 12,971 bucks) and was used as the pedigree matrix for genetic analyses. A total of 15 maximum generations and six complete generations were evaluated. Animals were born from June 1966 to November 2019. The linear appraisal was performed for 41,418 animals across the year. Animal records were collected from 76 farms in the south of Spain from 09/06/2010 to 18/12/2019. All the farms considered in the study had received official National and International Sanitary Certificates. All farms were controlled and officially declared tuberculosis-free (C3), brucellosis-free (M4) (Order of 22 June 2018 and Directive 91/68/EEC), and scrapie RC free [Regulation (EC) No 999/2001 of the European Parliament and the Council]. Additionally, these farms followed voluntary control plans for caprine contagious agalactia (CCA) (National CCA Surveillance, Control, and Eradication Programme 2018–2020) and caprine arthritis encephalitis (CAEV) (Order AYG/287/2019 of 28 February 2019). Goats were clinically examined by an official veterinarian, and individuals presenting signs of illness or disease were officially declared, removed from the herds, and discarded from the analyses. Permanent stabling practices were followed by all farms considered, and *ad libitum* water, forage, and supplemental concentrate were provided.

Records from 95 individuals were discarded due to missing or incomplete zoometric and linear appraisal observations. A total of 41,323 records, belonging to 22,727 herdbook-registered primipara does, 17,111 multipara does, and 1,485 bucks, were considered in the analysis. The average ages for primipara, multipara does, and bucks in the sample were 1.61 ± 0.35 years, 3.96 ± 1.74 years, and 2.43 ± 1.49 years (μ ± SD), respectively. Descriptive statistics, kurtosis, and skewness for Murciano-Granadina Linear appraisal system (LAS) zoometric traits can be found in Fernández Álvarez et al. (13).

#### 2.1.2. Murciano-Granadina linear appraisal system

Each observation comprises the rater’s score for each animal in the following four major categories for primipara and multipara does (three for bucks, young males, and yet-to-give-birth goats): structure and capacity, dairy structure, mammary system (except in males), and legs and aplomb. In primipara and multipara does, each record comprised information on 17 linear traits rated on a 9-point scale. Given that bucks were not scored for the mammary system major category, only 10 traits were scored for them following the aforementioned 9-point scale. Body depth from the structure and capacity major category and the dairy structure and legs and feet major categories followed the same criteria as the independence of sex and sexual status. The same trained rater scored all animals in the study.

Once all major categories are scored, the final score represents how close the overall animal comes to the optimal dairy standard. Murciano-Granadina LAS establishes that each major category contributes to the final score (25% for structure and capacity, 15% for dairy structure, 20% for legs and feet, and 40% for the mammary system) for primipara and multipara does (any doe that has ever produced milk). In bucks and young males, the percentages change to 50% for structure and capacity, 20% for dairy structure, and 30% for legs and fee.

The rater’s scores are assigned one of the six category qualifications considered by CAPRIGRAN as follows: insufficient (IN) for animals that display less than 69% of the optimal standard for Murciano-Granadina dairy goats (a final score of 69 points or less); mediocre (R), 70–74% of the optimal standard (a final score between 70 and 74 points); good (B), 75–79% of the optimal standard (a final score from 75 to 79 points); quite good (BB), 80–84% of the optimal standard (a final score from 80 to 84 points); very good (MB), 85–89% of the optimal standard (a final score from 85 to 89 points); or excellent (E), when at least 90% of the optimal standard is displayed (a final score higher than 90 points). The scales used and the translation process from zoometric traits to LAS traits is described in detail by Sánchez Rodríguez et al. (24) (Supplementary Table S1).

Age elements, for instance, the age of does and/or the lactation order condition the dairy linear or type appraisal-related traits (25). Hence, these elements, often considered and registered for does at appraisal, permit the adjustment of models for the outputs of linear or type appraisal records (26). The Pearson product–moment correlation coefficient between lactation stage and age in years was 0.705 (*p* < 0.01), hence redundancies could be presumed for the outputs of linear or type appraisal if both age components were simultaneously considered. Thus, the lactation stage was considered and results for primipara and multipara goats were reported separately.

#### 2.1.3. Preliminary assumption testing in zoometric and LAS traits

Common parametric assumptions were tested in the Murciano-Granadina goat breed zoometric and LAS historical records collected up until December 2019. A Kolmogórov–Smirnov test and Levene’s test were used to evaluate normality and homoscedasticity, respectively, using SPSS Statistics for Windows (version 25.0). Given the large sample size used in this study, the non-parametric method proposed by Hoeffding (27), which uses joint ranks, was chosen to test for the independence of two random variables with a continuous distribution function (df). To this aim, the Hmisc package’s hoeffd function (28) of RStudio 1.1.463 by the R Studio Team (29) was used. value of ps are approximated by linear interpolation on the table in Hollander et al. (30), which uses the asymptotically equivalent Blum–Kiefer–Rosenblatt statistic. For *p* < 0.0001 or > 0.5, *p-*values are computed using a well-fitting linear regression function in log P against the test statistic.

### 2.2. Genetic analyses

#### 2.2.1. Model and genetic parameter estimation for zoometric and LAS traits

The estimation of phenotypic and genetic parameters is presented, developed, and discussed in the study by Fernández Álvarez et al. (31). However, we summarized this process used for their estimation as follows.

The complete kinship matrix used for genetic analyses comprised all the 279,264 animals (266,793 does and 12,971 bucks) in the Murciano-Granadina goat breed pedigree. As the literature suggests, when bucks start rutting, male goats display behaviors associated with the urge to breed; they go through physical changes that even make specific variables, such as rump angle, decrease by 3 degrees (32). The breeding season for most goat breeds extends from August to January and they go into rut during Autumn (September, October, and November). The rut is characterized in bucks and the males of other species by an increase in testosterone, exacerbated sexual dimorphism, and increased aggression and interest in does (33). These cyclic changes over the year are the source of natural discrepancies in the definition and specific characteristics of zoometric traits between bucks and does, whose body changes are rather progressive during their lives across lactation stages. This, in turn, may lead to statistical biases; hence, we decided that the phenotype data set should only comprise those observations belonging to does, either primipara or multipara, when estimating genetic and phenotypic parameters.

As a result, a total of 39,838 records, belonging to 22,727 herdbook-registered primipara does and 17,111 multipara does, were considered in the genetic analysis. Animals were only scored once in their lifetime. Therefore, a multitrait animal mixed model with single measures was used to estimate (co) variance components, and the corresponding heritability, repeatability, phenotypic and genetic correlations, and standard errors of such correlations for the traits under examination. Pairs of two zoometric/linear appraisal variables were evaluated once each time until all possible pairwise combinations had been tested. In matrix notation, the following multitrait animal model with single measures was used:


$$\begin{array}{l} \mathrm{Yijklmn}=\mu +\mathrm{Fari}\cdot \mathrm{Ai}+\mathrm{LacStatj}\cdot \mathrm{Bj}+\mathrm{KMonk}\cdot \mathrm{Ck}\\ \;\;\;\;\;\;\;\;\;\;\;\;\;\;\;\;\;\;\;\;\;\;\;\;+\mathrm{IntFarmxKYearl}\cdot \mathrm{Dl}+b1\mathrm{DIMm}\cdot \mathrm{Em}\\ \;\;\;\;\;\;\;\;\;\;\;\;\;\;\;\;\;\;\;\;\;\;\;\;+b2\mathrm{An}\cdot \mathrm{Fn}+b_{3}^{2}A_{n}\cdot \mathrm{Fn}+\mathrm{eijklmn}, \end{array}$$

where Y_ijklmn_ is the vector of observations for each separate measure of each pair of zoometric or LAS traits (Supplementary Table S1) for a given animal; μ is the overall mean; Far_i_ is the vector for the fixed effect of the ith farm/herd (i = 76 farms); LacStatj is the vector for the fixed effect of the jth lactation stage (j = primipara/multipara does); KMon_k_ is the vector for the fixed effect of the kth kidding month (k = January to December); and IntFarm/KYear_l_ is the vector for the fixed effect of the lth level of interaction between farm/herd and kidding year (l = 400 interaction levels possibilities combining the 76 farms and kidding years from 2005 to 2019); days in milk was considered a linear covariate, hence b_1_ is the linear regression coefficient on days in milk (DIM_m_), age in years was considered a linear and quadratic covariate, hence b_2_ and $b_{3}^{2}$ are the linear and quadratic regression coefficients on the age of evaluation (A_n_), e_ijklmn_ is the vector of random residual effects, and A_i_, B_j_, C_k_, and D_l_ are incidence matrices relating records to their respective fixed effects, while E_m_ and F_n_ are incidence matrices relating records to their respective random effects. Only the direct genetic effect (animal) was fitted in each model due to zoometric/LAS scores being recorded only once for each animal.

The MTDFREML software package (34) was used to perform restricted maximum likelihood approach-based univariate analyses to compute heritabilities and variance components. The same software was used to carry out bivariate analyses to estimate covariates and genetic and phenotypic correlation. Genetic and phenotypic correlations between each individual conformation trait were estimated using a multivariate analysis including all traits. The iteration process used sought a convergence criterion level of 10^−12^. Link functions can be found in Boldman et al. (33). The standard errors for heritability and genetic and phenotypic correlations were computed using the same software.

As suggested by Navas González et al. (35), we used the phenotypical variance of each character and the existing phenotypical correlations between each possible pair combination for the estimation of the starting point to seek the convergence of additive genetic variance components (multiplying them by 0.2). Then, we did the same for environmental variances (multiplying them by 0.8) and genetic and phenotypic correlations to obtain specific variance components and estimates of fixed and random effects for each trait in multivariate analyses. To build the matrix of covariates among zoometric and LAS traits, the bivariate routine of the correlate procedure of the Analyze package in SPSS Statistics for Windows (version 25.0) was used. For this, users need to check the box next to cross-product deviations and covariances in the menu. Afterward, to obtain the covariance for each pairwise combination of variables, the sum of squares and cross-products must be divided by sample size (N).

### 2.3. Breeding value prediction (BLUPs, PBVs)

After convergence was reached, predicted breeding values were calculated through best linear unbiased predictors for random effects (BLUPs, PBVs) and their accuracies and reliabilities for zoometric and LAS traits for each animal in the matrix, using MTDFREML software. The standard errors for heritability and genetic and phenotypic correlations were computed by the same software. The fact that bucks were not considered for genetic evaluations and genetic parameter estimation must be considered. As a result, bucks’ breeding values were estimated from the female individuals connected to them through their genealogy. When the average difference in zoometric parameters between males and females is ignored, the estimate of heritability has been reported to reduce (36) as well, which may have also contributed to a reduction in BLUPs/PBVs.

#### 2.3.1. BLUP standard error of prediction (SEP), reliability (R_AP_), and accuracy (RTi)

Standard error of prediction (SEP), reliability (R_AP_), and accuracy (RTi) were calculated. The aforementioned parameters relate to each other through their definition and equation determination [R_AP_ = RTi^2^ = (1–SEP^2^/Va)^2^], from which Va is genetic additive variance.

First, reliability is the likelihood of someone repeating the experiment and getting the same result (repeatability), while accuracy measures how close a certain estimated value is to the real value. Their interpretation is, therefore, different. For example, for the evaluation of RTi, Scheme (37) suggests that less than 50% RTis mean PBVs are preliminary, thus calculated based on little information and hence very prone to change substantially as more direct information on the animal becomes available. RTis that range from 50 to 74% accuracy (medium) suggest that PBVs may have been calculated using the animal’s direct information and some limited indirect pedigree information. Medium/high RTis are denoted by 75–90% and may be calculated using the animal’s direct information together with the performance of a small number of its offspring. RTi values over 90% report estimates of the animal’s true breeding value, as it is unlikely that PBVs will change considerably even if additional information from offspring is added.

When reliability (R_AP_) is considered, the rule of thumb proposed by Horse (38) suggests that R_AP_ values less than 30% are generally unreliable, values between 30 and 55% are poor, values between 55 and 65% are sufficient, values between 65 and 75% are more than sufficient, values between 75 and 90% are good, and values higher than 90% are very reliable and repeatable; values around 60% suggest the information strongly relies on offspring information, which would be undesirable.

Last but not least, the standard error of prediction (SEP) measures how large prediction errors (residuals) are for the data set measured in the same unit for each of the zoometric or LAS traits measured; therefore, it provides a direct measure of possible change, i.e., the risk of the true breeding value of the animal (TBV) not to be aligned on the PBV.

Van Vleck (39) suggested that possible change is the risk in units of the trait breeding value not being true and can be “positive” or “negative.” This means the likelihood of true BV being higher than PBV by a certain amount (possible gain) is the same as the likelihood of the true BV being lower than the PBV by the same amount (possible loss). Contextually, confidence intervals are normally used to determine the likelihood of possible change assuming a normal distribution of the TBV around the PBV.

The first half of the TBV would be expected to be higher than the PBV, while the second would be expected to be lower than the PBV. The interval range from PBV – (1) SEP to PBV + (1) SEP corresponds to a 68% probability that the TBV for an animal is centered on the PBV for the animal. Such an interval can be narrowed or widened corresponding to the probability of the TBV in the interval. For instance, one could expect the interval from PBV – (2) SEP to PBV + (2) SEP to contain 95% of the TBV. Units of SEP other than (1) or (2) would correspond to other confidence intervals. With a 68% confidence interval, 32% would be half over and half below the intervals’ ends, while with the 95% interval, the percentage placed out of the interval would be 5% (again half over and half below each end). Ranges for many combinations of PBV and SEP may overlap considerably. Then, by observing which PBV centers the interval and comparing intervals, a rather direct measure of risk compared with that of accuracy (RTi) is obtained.

#### 2.3.2. Descriptive statistics and differences in zoometric/linear appraisal PBVs across casein haplotypes

Maximum and minimum for zoometric/linear appraisal traits predicted breeding values (PBVs) and standard error of prediction (SEP). Accuracy (RTi) and reliability (R_AP_) were calculated using the *Descriptive statistics* routine of the *Analyze* set of SPSS (version 26.0). Afterward, a Kruskal–Wallis H test was used to study the potential existing differences between predicted breeding values for zoometric/linear appraisal traits across haplotypes of the same casein gene, as three or more possibilities were available using the *independent samples* routine of the *Nonparametric tests* package within the *Analyze* set of SPSS (version 26.0).

#### 2.3.3. Casein haplotyping

##### 2.3.3.1. Haplotyping animal samples and the selection process

Given the costs involved in genotyping, a selection process of goats that had been considered for milk yield standardization and composition analysis was implemented. This sample selection process aimed at genotyping a representative sample of animals for 48 SNPs in the casein complex from which complete records for several lactations existed. Hence, animals present in the herdbook of the National Association of Breeders of Goats of the Murciano-Granadina breed (CAPRIGRAN) were ranked considering the most recent and updated official breeding values for milk yield and composition reported for all the animals published in 2015. Provided multiple traits were considered, we developed combined selection index (ICO) procedures following the premises in Van Vleck (40) to summarize the value of each individual comprising each of its partial values for milk yield and composition; these procedures were computed for each animal using MatLab r2015a (41). We decided not to include the dry matter in the ICO, as redundancies may occur deriving from the relationship between this trait and fat or protein content. To determine the weights to apply to each trait, we considered the phenotypic relationship across milk yield and composition traits (except for dry matter), scoring their relevance as selection criteria when the breeding goal was milk yield and quality. In matrix notation, the weights to be applied on the selection index combining the partial scores of each modality were obtained as $b=P^{-1}g$ , where b is the vector of the weights to be applied to each production or content trait, P is the phenotypic (co)variance matrix, and g is the vector of genetic (co)variances of every trait with each other. As a result and considering the market demands, the weights for milk yield, fat, protein, and lactose followed the proportion of 1:1:1:1, respectively. The combined index used (ICO) was as follows:


$$\mathrm{ICO}=\frac{{\mathrm{PBV}}_{\mathrm{milkyield}}W_{1}}{\mu_{\mathrm{milkyield}}}+\frac{{\mathrm{PBV}}_{\mathrm{fat}}W_{2}}{\mu_{\mathrm{fat}}}+\frac{{\mathrm{PBV}}_{\mathrm{protein}}W_{3}}{\mu_{\mathrm{protein}}}+\frac{{\mathrm{PBV}}_{\mathrm{lactose}}W_{4}}{\mu_{\mathrm{lactose}}},$$

where PBV is the predicted breeding value for each of the traits and animals included in the matrix; W_1_ is the weight for milk yield, W_2_ is the weight for fat, W_3_ is the weight for protein, and W_4_ is the weight for lactose in kg and normalized at 210 days; and μ is the mean for each of the traits included in the ICO computed in kg and at 210 days. After the ICO was computed for each of the animals included in the matrix, we sorted a total of 200 animals from the whole routine milk recording of the Murciano-Granadina goat breed in a ranking considering their ICO value obtained at the previous genetic evaluation. Animals with extreme PBVs may be less efficient and less balanced than expected at first. Furthermore, not all traits are affected to the same degree by selection for these extremes. For these reasons, 200 animals were ranked as follows: we chose 67 females presenting the lowest ICO values in the rank, 66 females with values around percentile 50, and 67 females presenting the highest ICO values in the rank to perform an adjusted representative sampling of the genotype distribution in the population. The samples belonging to animals with missing or incomplete phenotype registries were discarded, hence the final set for genotyping consisted of blood samples from 108 stud book-registered goats out of the 200 animals that were initially selected. The records were collected from 28 southern Spanish farms, the records of which were collected in random periods from October 2006 to June 2018. The mean age of the animals in the sample was 1.39 years old (from 1 to 9.15 years).

#### 2.3.4. Genotyping and linkage disequilibrium

A modification of the procedure described by Miller et al. (42) was performed for DNA isolation. To this aim, 16 non-related does were randomly chosen from the herdbook of the breed. Oligonucleotide sequences, SNP promoters, UTRH3’ regions, and polymorphic exons have been described by Pizarro Inostroza et al. (43). A Platinum High Fidelity (LifeTechnology, CA) PCR kit was used to amplify polymorphic regions. MACROGEN sequencing service (Macrogen Inc., Korea) sequenced the PCR product, and MEGA7 software and Ensembl Genome Browser 97 database were used to analyze pherograms and evaluate previous annotations for SNPs (44). Genotyping was performed using a KASP assay (LGC Limited, Fordham, United Kingdom) and KlusterCaller software (LGC Limited, Fordham, United Kingdom). Heterozygosity values of approximately 40% suggested that the number of SNPs used as genomic controls was sufficient (45) to prevent the effects of population stratification.

Minor allele frequency (MAF) was calculated to differentiate between common and rare variants (MAF < 0.05) using PLINK v1.90 (46). The linkage disequilibrium extent (LD) of casein complex SNPs was calculated using HaploView software (11), scoring LD through D′ (normalized linkage disequilibrium coefficient) and r2 (linkage disequilibrium coefficient of determination) (Supplementary Table S2). The total length of casein loci and distances between adjacent loci were determined following the premises presented by Dagnachew et al. (47).

#### 2.3.5. Haplotyping

Phasing (or haplotyping) describes the process of determining haplotypes from the genotype data (47). As suggested by Glusman et al. (48), haplotypes are more specific than less complex variants such as single nucleotide variants (SNP variants). A haplotype-based empirical model inherited from an SNP-based method was followed as suggested by Chen et al. (49). We identified 48 single nucleotide polymorphisms (SNPs) present in the casein complex of 159 unrelated individuals of diverse ancestry, which organized the SNPs into 86 different haplotypes. Haplotype sequences and the maximum and minimum values for the predicted breeding values of each zoometric/linear appraisal trait are shown in Supplementary Table S3. The results from the analyses of epistatic relationships in Pizarro Inostroza et al. (50) were also considered for validating haplotyping.

### 2.4. Canonical discriminant analysis

If protein levels in milk have been associated to certain zoometric variables at a phenotypic level, it is reasonable to think that a genetic connection may exist between such zoometric traits and specific regions in the genome encoding for the expression of some of those proteins in milk. Haplotypes act as categorical variables, while PBVs act as numeric traits, hence DCA is appropriate for testing differences exhaustively. Canonical discriminant analyses (CDAs) were performed to design a tool that enables the classification of goats while determining whether linear combinations of predicted breeding values for zoometric/linear appraisal traits [stature (height to withers), rump width, rear insertion height, rump angle, angulosity, chest width, udder width, nipple placement, nipple diameter, bone quality, anterior insertion, median suspensor ligament, mobility, body depth, udder depth, rear legs side view, and rear legs rear view] describe within-and between-population αS1, αS2, β, and κ casein haplotypes and haplogroups clustering patterns. The explanatory variables used for the present analyses were the predicted breeding values for each of the zoometric/linear appraisal-related traits presented in Supplementary Table S1. The haplotype and haplogroups for each of the four caseins (αS1, αS2, β, and κ) were considered the clustering criterion.

Canonical relationships with traits were plotted to depict the group differences in an easily interpretable territorial map. Regularized forward stepwise multinomial logistic regression algorithms were used to perform the variable selection. Priors were regularized according to the group sizes calculated using the prior probability of commercial software (SPSS Version 26.0 for Windows, SPSS, Inc., Chicago, IL) instead of considering them the same to avoid groups with different sample sizes affecting the quality of the classification (51).

The same sample size contexts as those used in this study across groups have been reported to be robust. In this regard, some authors have reported a minimum sample size of at least 20 observations for every four or five predictors, and the maximum number of independent variables should be n-2, where n is the sample size, to palliate possible distortion effects (51, 52). Consequently, the present study used a four or five times higher ratio between observations and independent variables than those described above, which renders discriminant approaches efficient. Multicollinearity analysis was run to ensure independence and a strong linear relationship across predictors. Variables chosen by the forward or backward stepwise selection methods were the same. Finally, the progressive forward selection method was performed since it requires less time than the backward selection method.

The discriminant routine of the Classify package of SPSS (version 26.0) and the canonical discriminant analysis routine of the Analyzing Data package of XLSTAT (Addinsoft Pearson Edition 2014, Addinsoft, Paris, France) were used to perform canonical discriminant analysis.

#### 2.4.1. Multicollinearity preliminary testing

Multicollinearity refers to the linear relationship between two or more variables, which also means a lack of orthogonality between them. Different methods are available to detect multicollinearity, and the most widely used are variance inflation factor (VIF) and tolerance (53). VIF is a ratio of variance in a regression model with multiple attributes divided by the variance of a model with only one attribute (54). Explained more technically and exactly, multicollinearity occurs when k vectors lie in a subspace of dimension less than k. Multicollinearity can explain a data-poor condition, which is frequently found in observational studies in which the researchers do not interfere with the study. Thus, many investigators often confuse multicollinearity with correlation. However, correlation is the linear relationship between just two variables; multicollinearity can exist between two variables or between one variable and the linear combination of the others. Therefore, correlation is considered a special case of multicollinearity. A high correlation implies multicollinearity, but not the other way around. Before performing the statistical analyses *per se*, a multicollinearity analysis was run to discard potentially strong linear relationships across explanatory variables and ensure data independence. In this way, before data manipulation, redundancy problems can be detected, which limits the effects of data noise and reduces the error term of discriminant models. The multicollinearity preliminary test helps to identify unnecessary variables that should be excluded, preventing the overinflation of variance explanatory potential and type II error increase (55). The variance inflation factor (VIF) was used to determine the occurrence of multicollinearity issues. The literature reports a recommended maximum VIF value of 5 (56). On the other hand, tolerance (1 − R^2^) concerns the amount of variability in a certain independent variable that is not explained by the rest of the dependent variables considered (tolerance >0.20) (57). The multicollinearity statistics routine of the describing data package of XLSTAT (Addinsoft Pearson Edition 2021, Addinsoft, Paris, France) was used. The following formula was used to calculate the VIF:


$$\mathrm{VIF}=1/\left(1-R^{2}\right),$$

where R^2^ is the coefficient of determination of the regression equation.

#### 2.4.2. Canonical correlation dimension determination

The maximum number of canonical correlations between two sets of variables is the number of variables in the smaller set. The first canonical correlation usually explains most of the relationships between different sets. In any case, attention should be given to all canonical correlations, despite reporting only the first dimension as common in previous studies (58). When canonical correlation values are 0.30 or higher, they correspond to approximately 10% of the variance explained.

#### 2.4.3. Canonical correlation analysis efficiency

**Figure 1:**
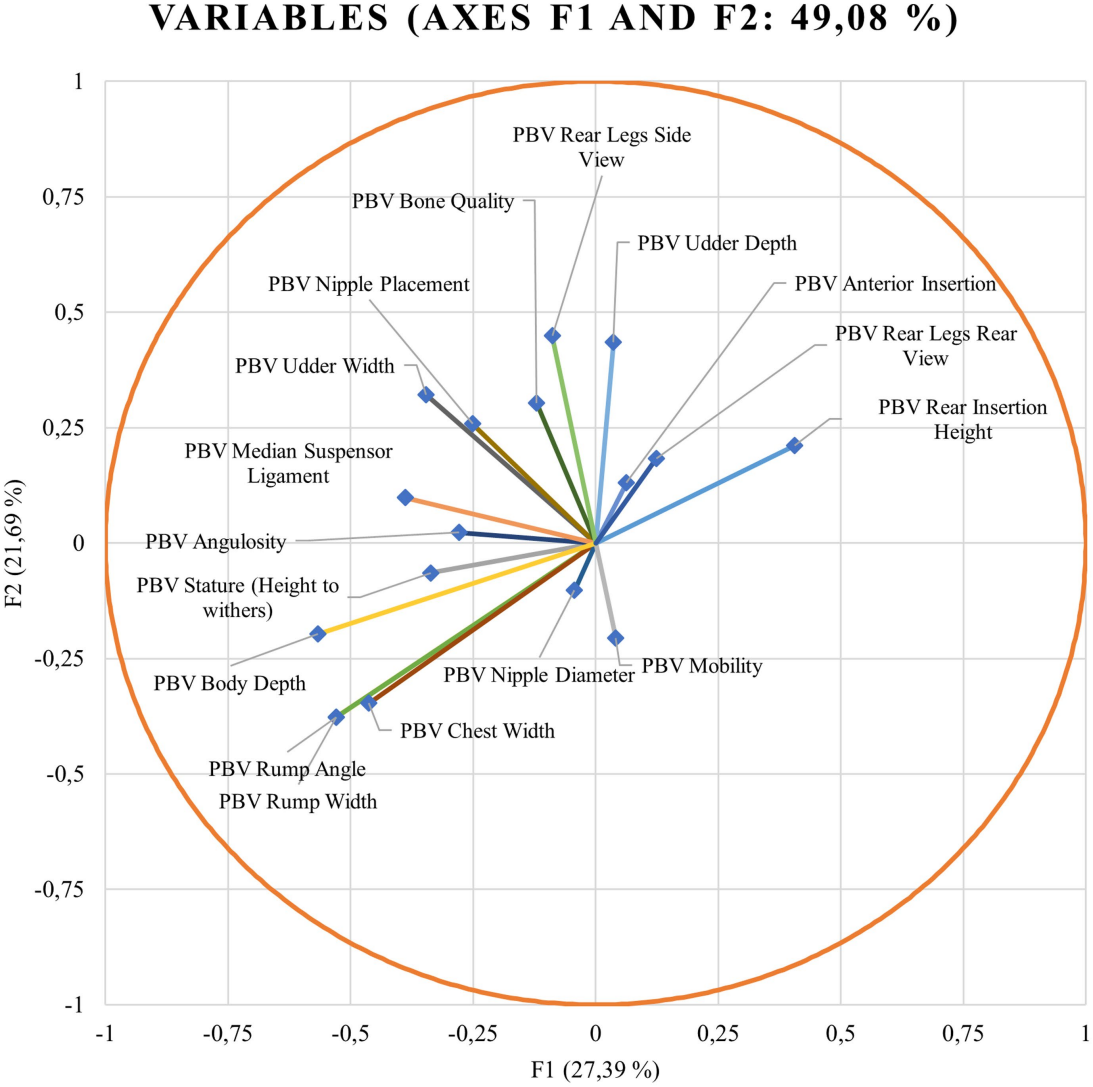
Discriminant loadings for the predicted breeding values (PBVs) for zoometrics/linear appraisal traits determining the relative weight of each trait on each canonical discriminant function.

Wilks’ lambda test evaluates which variables may significantly contribute to the discriminant function. When Wilks’ lambda approximates 0, the contribution of that variable to the discriminant function increases. χ^2^-tests the Wilks’ lambda significance. If the significance is below 0.05, it can be concluded that the function can explain the group adscription well (59). Discriminant loadings for predicted breeding values for zoometric/linear appraisal traits determining the relative weight of each trait on each canonical discriminant function are shown in Figure 1.

#### 2.4.4. Canonical discriminant analysis model reliability

Pillai’s trace criterion, as the only acceptable test to be used in cases of unequal sample sizes, was used to test the assumption of equal covariance matrices in the discriminant function analysis (60). Pillai’s trace criterion was computed as a subroutine of the canonical discriminant analysis routine of the Analyzing Data package of XLSTAT (Addinsoft Pearson Edition 2014, Addinsoft, Paris, France). A significance of ≤0.05 is indicative of the set of predictors considered in the discriminant model being statistically significant. Pillai’s trace criterion is argued to be the most robust statistic for general protection against departures from the multivariate residuals’ normality and homogeneity of variance. The higher the observed value for Pillai’s trace is, the stronger the evidence that the set of predictors has a statistically significant effect on the values of the response variable, i.e., Pillai’s trace criterion shows potentially linear differences in the predicted breeding values for zoometric/linear appraisal traits across β casein haplotype clustering groups (61).

#### 2.4.5. Canonical coefficients, loading interpretation, and spatial representation

When CDA is implemented, a preliminary principal component analysis is used to reduce the overall variables into a few meaningful variables that contributed most to the variations across haplotypes. The use of the CDA determined the percentage assignment of does within their casein haplotypic group. Variables with a discriminant loading of ≥|0.40| were considered substantive, indicating substantive discriminating variables. The stepwise procedure technique was used to prevent non-significant variables entering the function. Coefficients with large absolute values correspond to variables with greater discriminating ability. Data were standardized following procedures reported by Manly and Alberto (62). Then, squared Mahalanobis distances and principal component analysis were computed using the following formula:


$$D_{\mathrm{ij}}^{2}=\left({\bar{ϒ}}_{i}-{\bar{ϒ}}_{j}\right)\;{\mathrm{COV}}^{-1}\left({\bar{ϒ}}_{i}-{\bar{ϒ}}_{j}\right),$$

where $D_{\mathrm{ij}}^{2}$ is the distance between population i and j; COV^−1^ is the inverse of the covariance matrix of measured variable x; and ${\bar{ϒ}}_{i}$ and ${\bar{ϒ}}_{j}$ are the means of variable x in the ith and jth populations, respectively.

The squared Mahalanobis distance matrix was converted into a Euclidean distance matrix, and a dendrogram was built using the underweighted pair-group method arithmetic averages (UPGMA; Rovira i Virgili University, Tarragona, Spain) and the phylogeny procedure of MEGA X 10.0.5 (Institute of Molecular Evolutionary Genetics, The Pennsylvania State University, State College, PA, United States).

#### 2.4.6. Discriminant function cross validation

Afterward, to determine the probability that a goat presenting an unknown haplotype belongs to a particular haplotypic group (63), the hit ratio parameter was computed. For this, the relative distance of the problem observation to the centroid of its closest group was used. The hit ratio is the percentage of goats that are correctly ascribed to the casein haplotype form that they present. A leave-one-out cross-validation procedure is used as a form of significance to consider whether the discriminant functions can be validated. Classification accuracy is achieved when the classification rate is at least 25% higher than that obtained by chance.

Press’ Q statistic can support these results as it can be used to compare the discriminating power of the cross-validated function, as follows:


$$\mathrm{Press}\;Q^{\prime }=\frac{{\left[n-\left(n\prime K\right)\right]}^{2}}{n\left(K-1\right)},$$

where n is the number of observations in the sample; n’ is the number of observations correctly classified; and K is the number of groups.

The value of Press’ Q statistic must be compared with the critical value of 6.63 for χ^2^ with a degree of freedom at a significance of 0.01. When Press’ Q exceeds the critical value of χ^2^ = 6.63, the cross-validated classification can be regarded as significantly better than chance.

## 3. Results

### 3.1. Genetic parameters estimation, breeding value prediction, and comparative descriptive analysis

The estimation of genetic parameters was performed as a necessary intermediate stage for the prediction of breeding values. The estimation, presentation of results, and a deep discussion can be accessed in Fernández Álvarez et al. (31). A summary of the maximum and minimum predicted breeding values (PBV), standard error of prediction (SEP), accuracy (RTi), and reliability (R_AP_) for zoometrics and LAS traits sorted by sex and lactation stage is shown in Table 1. Maximum and minimum PBVs for almost all traits were slightly to moderately higher in bucks, except for stature [height to withers, anterior insertion, and nipple diameter, which otherwise reported the broadest ranges for R_AP_ in bucks (0.000–0.980) when compared to primipara (0.000–0.672) or multipara does (0.000–0.740)]. The lowest R_AP_ was reported for the PBVs for zoometric or LAS traits in either multipara or primipara does, while the highest was again reported for stature (height to withers, anterior insertion, and nipple diameter in bucks).

**Table 1:** Minimum and maximum predicted breeding values and standard error of prediction (SEP).

			Bucks		Primipara		Multipara	
Major area	Zoometric/LAS trait	Parameter	Minimum	Maximum	Minimum	Maximum	Minimum	Maximum
Structure and capacity	Stature (height to withers)	PBV	−1.862	1.814	−1.850	2.513	−2.076	2.548
		SEP	0.110	0.790	0.400	0.720	0.350	0.750
		RAP	0.000	0.980	0.000	0.672	0.000	0.740
		Rti	0.000	0.990	0.000	0.820	0.000	0.860
	Chest width	PBV	−1.158	1.589	−1.089	1.382	−1.370	1.373
		SEP	0.110	0.620	0.370	0.570	0.320	0.590
		RAP	0.000	0.960	0.000	0.593	0.000	0.672
		Rti	0.000	0.980	0.000	0.770	0.000	0.820
	Body depth	PBV	−0.697	0.680	−0.486	0.506	−0.661	0.581
		SEP	0.080	0.290	0.210	0.270	0.190	0.270
		RAP	0.000	0.903	0.000	0.436	0.000	0.490
		Rti	0.000	0.950	0.000	0.660	0.000	0.700
	Rump width	PBV	−0.838	0.911	−0.924	0.826	−1.024	0.911
		SEP	0.070	0.410	0.240	0.380	0.210	0.390
		RAP	0.000	0.960	0.000	0.608	0.000	0.689
		Rti	0.000	0.980	0.000	0.780	0.000	0.830
	Rump angle	PBV	−0.693	0.886	−0.597	0.713	−0.862	0.775
		SEP	0.080	0.370	0.250	0.340	0.220	0.350
		RAP	0.000	0.941	0.000	0.504	0.000	0.578
		Rti	0.000	0.970	0.000	0.710	0.000	0.760
Dairy structure	Angulosity	PBV	−1.194	1.504	−1.351	1.184	−1.239	1.295
		SEP	0.110	0.580	0.360	0.530	0.310	0.550
		RAP	0.000	0.960	0.000	0.578	0.000	0.640
		Rti	0.000	0.980	0.000	0.760	0.000	0.800
	Bone quality	PBV	−1.418	1.206	−1.131	1.060	−1.167	1.170
		SEP	0.070	0.430	0.250	0.390	0.220	0.410
		RAP	0.000	0.960	0.000	0.608	0.000	0.689
		Rti	0.000	0.980	0.000	0.780	0.000	0.830
Mammary system	Anterior insertion	PBV	−1.937	2.690	−1.676	2.418	−2.083	2.763
		SEP	0.150	1.050	0.550	0.960	0.480	0.990
		RAP	0.000	0.980	0.000	0.656	0.000	0.740
		Rti	0.000	0.990	0.000	0.810	0.000	0.860
	Rear insertion height	PBV	−0.949	1.048	−0.958	0.798	−1.059	1.172
		SEP	0.080	0.460	0.290	0.420	0.250	0.440
		RAP	0.000	0.960	0.000	0.563	0.000	0.640
		Rti	0.000	0.980	0.000	0.750	0.000	0.800
	Median suspensor ligament	PBV	−1.201	1.688	−1.138	1.281	−1.390	1.556
		SEP	0.110	0.690	0.390	0.630	0.340	0.650
		RAP	0.000	0.960	0.000	0.608	0.000	0.689
		Rti	0.000	0.980	0.000	0.780	0.000	0.830
	Udder width	PBV	−0.545	0.592	−0.429	0.487	−0.435	0.497
		SEP	0.070	0.260	0.180	0.240	0.170	0.240
		RAP	0.000	0.903	0.000	0.423	0.000	0.490
		Rti	0.000	0.950	0.000	0.650	0.000	0.700
	Udder depth	PBV	−1.288	2.001	−1.165	1.441	−1.970	1.761
		SEP	0.000	0.710	0.000	0.650	0.000	0.670
		RAP	0.000	0.960	0.000	0.593	0.000	0.672
		Rti	0.000	0.980	0.000	0.770	0.000	0.820
	Nipple placement	PBV	−0.781	1.056	−0.939	0.685	−0.955	0.953
		SEP	0.080	0.440	0.270	0.400	0.230	0.420
		RAP	0.000	0.960	0.000	0.578	0.000	0.656
		Rti	0.000	0.980	0.000	0.760	0.000	0.810
	Nipple diameter	PBV	−1.940	2.691	−1.668	2.405	−2.097	2.768
		SEP	0.150	1.050	0.550	0.960	0.480	0.990
		RAP	0.000	0.980	0.000	0.656	0.000	0.740
		Rti	0.000	0.990	0.000	0.810	0.000	0.860
Legs aplomb	Rear legs rear view	PBV	−0.735	0.643	−1.096	0.547	−0.981	0.589
		SEP	0.060	0.330	0.210	0.300	0.190	0.310
		RAP	0.000	0.960	0.000	0.548	0.000	0.608
		Rti	0.000	0.980	0.000	0.740	0.000	0.780
	Rear legs side view	PBV	−0.389	0.385	−0.366	0.248	−0.360	0.376
		SEP	0.060	0.220	0.160	0.210	0.150	0.210
		RAP	0.000	0.903	0.000	0.410	0.000	0.476
		Rti	0.000	0.950	0.000	0.640	0.000	0.690
	Mobility	PBV	−0.488	0.536	−0.357	0.375	−0.478	0.511
		SEP	0.060	0.220	0.160	0.210	0.140	0.210
		RAP	0.000	0.922	0.000	0.436	0.000	0.504
		Rti	0.000	0.960	0.000	0.660	0.000	0.710

Accuracy (RTi) and reliability (R_AP_) of zoometric and LAS traits obtained in bivariate analyses through REML methods sorted by sex and lactation stage.

The RTi scores confirmed a similar pattern, and the Pearson product–moment correlation analysis revealed a correlation coefficient of approximately 1, indicating a high level of consistency in the genetic parameters between the PBVs for zoometric and LAS traits. Additionally, this correlation was statistically significant (*p* < 0.001), suggesting that the translation of zoometry to LAS was nearly flawless.

### 3.2. Differences in zoometric/linear appraisal trait predicted breeding values across casein haplotypes

The only significant differences revealed after the performance of the Kruskall–Wallis H test (*p* < 0.05) were found across the haplotypes of the β casein gene for the predicted breeding values of stature (height to withers), rump width, rump angle, median suspensor ligament, and body depth.

### 3.3. Canonical discriminant analysis model reliability

**Figure 2:**
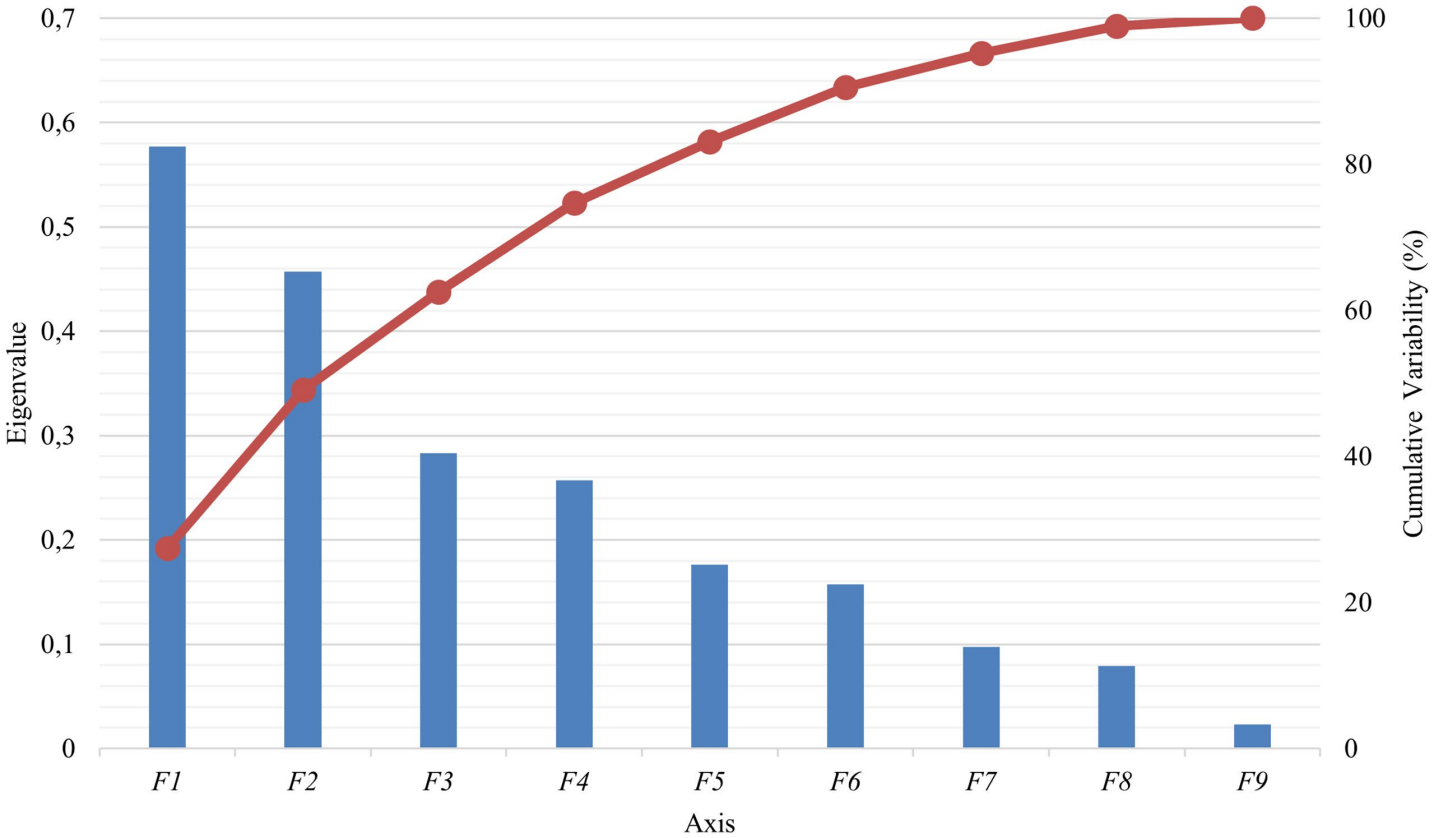
Canonical variable functions and percentages of self-explained and cumulative variance for β casein.

PBVs for stature (height to withers) and rump width were discarded from the analyses because they presented VIF values over 5 (Table 2). A significant Pillai’s trace criterion determined that discriminant canonical analysis was only feasible in β casein (Table 3). As reported in Table 4, only one out of the nine discriminant functions designed after the analyses presented a significant discriminant ability. The discriminatory power of the F1 function was high (eigenvalue of 0.5773; Figure 2), with approximately 50% of the variance explained by F1 and F2.

**Table 2:** Multicollinearity analysis of predicted breeding values for zoometric/linear appraisal traits in Murciano-Granadina goats.

αS2 casein	Tolerance	VIF	αS1 casein	Tolerance	VIF	β casein	Tolerance	VIF	κ casein	Tolerance	VIF
PBV Nipple diameter	0.85	1.18	PBV nipple diameter	0.78	1.28	PBV rear legs side view	0.81	1.23	PBV nipple diameter	0.82	1.23
PBV rear legs side view	0.81	1.23	PBV rear legs side view	0.75	1.33	PBV nipple diameter	0.80	1.25	PBV rear legs side view	0.79	1.26
PBV median suspensor ligament	0.80	1.26	PBV median suspensor ligament	0.72	1.38	PBV median suspensor ligament	0.76	1.32	PBV median suspensor ligament	0.78	1.29
PBV udder depth	0.71	1.41	PBV udder depth	0.66	1.52	PBV udder depth	0.65	1.53	PBV udder depth	0.61	1.63
PBV mobility	0.60	1.68	PBV nipple placement	0.60	1.66	PBV nipple placement	0.60	1.67	PBV mobility	0.61	1.64
PBV nipple placement	0.59	1.68	PBV mobility	0.57	1.74	PBV mobility	0.58	1.74	PBV body depth	0.55	1.83
PBV body depth	0.58	1.73	PBV body depth	0.54	1.84	PBV body depth	0.57	1.74	PBV angulosity	0.54	1.85
PBV angulosity	0.57	1.75	PBV angulosity	0.54	1.85	PBV angulosity	0.55	1.81	PBV anterior insertion	0.54	1.86
PBV anterior insertion	0.54	1.86	PBV anterior insertion	0.48	2.06	PBV anterior insertion	0.53	1.90	PBV nipple placement	0.53	1.90
PBV bone quality	0.48	2.07	PBV bone quality	0.48	2.07	PBV bone quality	0.49	2.05	PBV bone quality	0.50	2.00
PBV udder width	0.46	2.17	PBV rear legs rear view	0.43	2.32	PBV udder width	0.44	2.25	PBV rear legs rear view	0.48	2.08
PBV rear insertion height	0.44	2.28	PBV udder width	0.43	2.33	PBV rear legs rear view	0.43	2.31	PBV Rear insertion height	0.47	2.14
PBV rear legs rear view	0.41	2.43	PBV rear insertion height	0.38	2.67	PBV rear insertion height	0.43	2.34	PBV udder width	0.46	2.17
PBV rump width	0.30	3.36	PBV rump width	0.29	3.47	PBV rump width	0.29	3.40	PBV rump width	0.30	3.28
PBV chest width	0.28	3.60	PBV chest width	0.25	3.98	PBV chest width	0.27	3.74	PBV chest width	0.27	3.77

Interpretation thumb rule: VIF = 1 (not correlated); 1 < VIF < 5 (moderately correlated); VIF ≥ 5 (highly correlated).

PBV, predicted breeding value; tolerance, 1 – R^2^.

**Table 3:** Pillai’s trace criterion for predicted breeding values for zoometrics/linear appraisal traits across casein haplotypes in Murciano-Granadina goats.

Statistics/haplotypes	αS2 casein	αS1 casein	β casein	κ casein
Pillai’s trace criterion	1.441	1.017	1.506	1.734
F (Observed value)	0.914	0.854	1.220	1.023
F (Critical value)	1.207	1.246	1.230	1.209
DF1	165.000	120.000	135.000	165.000
DF2	1001.000	704.000	819.000	902.000
*p*-value	0.763	0.858	0.050	0.415

**Table 4:** Canonical discriminant analysis efficiency parameters for determining the significance of each canonical discriminant function.

Test of function(s)	Wilks’ lambda	Chi-square	df	*p*-value
1 through 9	0.18	161.67	135	0.05
2 through 9	0.28	119.25	112	0.30
3 through 9	0.40	85.50	91	0.64
4 through 9	0.51	62.47	72	0.78
5 through 9	0.65	41.07	55	0.92
6 through 9	0.75	26.43	40	0.95
7 through 9	0.84	16.33	27	0.95
8 through 9	0.92	8.17	16	0.94
9 through 9	0.98	1.87	7	0.97

df, degrees of freedom.

### 3.4. Canonical coefficients, loading interpretation, and spatial representation

Variables were ranked depending on their discriminating properties. For this, a test of equality of group means across β casein haplotypes was used (Table 5). Lower values of Wilks’ lambda and greater values of F indicate a better discriminating power, which translates into a better position in the rank.

**Table 5:** Results of the tests of equality of the β casein haplotype group means to test for differences in the means across Murciano-Granadina goats once redundant variables have been removed.

Predicted breeding value	Wilks’ lambda	*F*	df1	df2	*p*-value	Rank
Stature (height to withers)	0.87	1.65	9	97	0.11	NS
*Rump width*	*0.81*	*2.56*	*9*	*97*	*0.01*	*1*
Rear insertion height	0.87	1.58	9	97	0.13	NS
Rump angle			9	97		NS
Angulosity	0.94	0.74	9	97	0.67	NS
*Chest width*	*0.85*	*1.92*	*9*	*97*	*0.05*	*4*
Udder width	0.87	1.57	9	97	0.13	NS
Nipple placement	0.87	1.67	9	97	0.11	NS
Nipple diameter	0.94	0.70	9	97	0.71	NS
Bone quality	0.89	1.34	9	97	0.22	NS
Anterior insertion	0.92	1.00	9	97	0.44	NS
*Median suspensor ligament*	*0.83*	*2.19*	*9*	*97*	*0.03*	*3*
Mobility	0.92	0.89	9	97	0.54	NS
*Body depth*	*0.82*	*2.39*	*9*	*97*	*0.02*	*2*
Udder depth	0.87	1.60	9	97	0.13	NS
Rear legs side view	0.88	1.41	9	97	0.19	NS
Rear legs rear view	0.88	1.47	9	97	0.17	NS

NS, non-significant.

**Figure 3:**
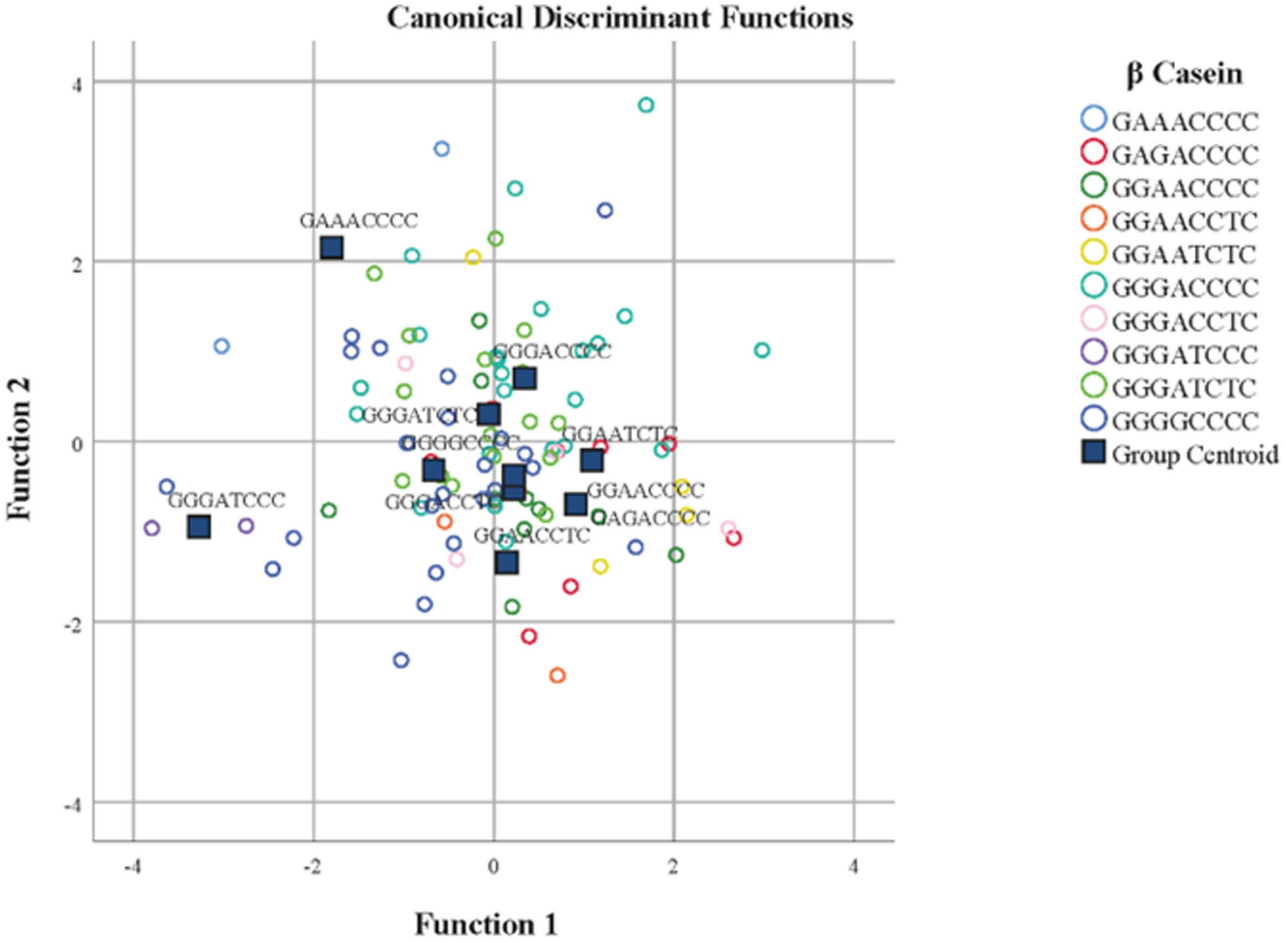
Territorial map depicting the goats considered in the canonical discriminant analysis sorted across β casein in Murciano-Granadina goats.

**Figure 4:**
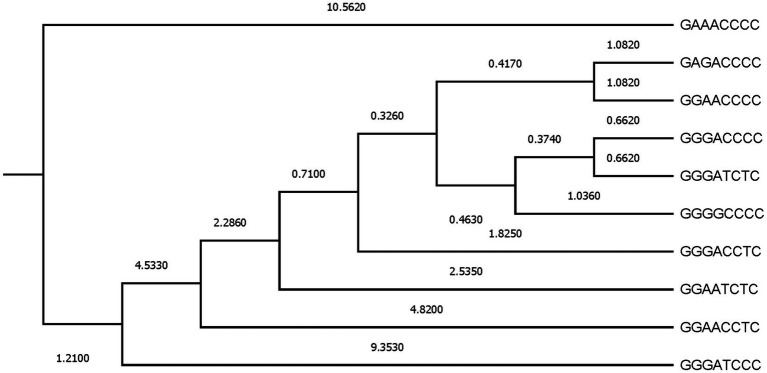
Dendogram constructed from Mahalanobis distances across β casein haplotypes.

Standardized discriminant coefficients measure the relative weight of each predicted breeding value for zoometric/linear appraisal traits in the discriminant functions (Figures 1, 3). Out of the nine significant discriminant functions (Table 4), only the two most relevant functions were used to build a standardized discriminant coefficient biplot, capturing the highest fraction of variance (Figure 1). In this regard, those variables that have a vector extending further apart from the origin most relevantly contributed to the first (F1) and second (F2) discriminant functions. Figures 3, 4 suggest clear differentiation among Murciano-Granadina goats across the β casein haplotypes considered in the analyses. The relative position of centroids was determined through the substitution of the mean value for observations in each term of the first two discriminant functions (F1 and F2). The larger the distance between centroids, the better the predictive power of the canonical discriminant function in classifying observations. Supplementary Tables S2, S3 report the results obtained in the classification and leave-one-out cross-validation. A Press’ *Q*-value of 210.19 (*N* = 108; *n* = 56; *K* = 10) was obtained. Therefore, it can be considered that predictions were significantly better than chance at 95% (64).

Additionally, to evaluate the proximity between β casein haplotypes, Mahalanobis distances were represented (Figure 4). Two main clusters were formed, the first represented by GAAACCCC, which was the most distant haplotype from the rest (Mahalanobis distance of 10.5620) when zoometrics/linear appraisal predicted breeding values were considered and the second subcluster comprising the nine remaining β casein haplotypes. A progressive segregation of haplotypes occurred within the second cluster, first derived from the changes from G→A and from T→C at the third and fifth SNP positions in the β casein haplotype, and second, derived from the change back to the former position of C→T at the fifth SNP in the β casein haplotype. The third segregation step undid the changes from G→A and from T→C at the third and fifth SNP positions in the β casein haplotype.

Afterward, a complex fourth cluster was formed that presented two main branches. The first one developed around the presence of GGG at the first, second, and third positions in the β casein haplotype, and the second one was based upon the alternating change from G→A at the second and third SNPs within the β casein haplotype and the change of C→T, even if the latter did not appear to be a source for β casein haplotype differences.

As denoted by Figures 3, 4, the results in the territorial map depicting the goats considered in the canonical discriminant analysis sorted across β casein in Murciano-Granadina goats suggest the extreme possibilities may be marked by the haplotypic sequences GGAACCCC and GGGATCCC, which was also revealed in Supplementary Table S2, with GGAACCCC reporting the largest maximum values for predicted breeding values for zoometric/linear appraisal traits, while the lowest maximum values were reported for GGGATCCC. GGAACCCC reported positive maximum predicted breeding values for all traits, while the opposite situation was described by GGGATCCC, for which negative maximum predicted breeding values were reported for all zoometric/linear appraisal traits except rear insertion height, bone quality, anterior insertion, udder depth, rear legs side view, and rear legs rear view.

## 4. Discussion

To maintain the quality and productivity of the Murciano-Granadina breed, it is essential to carefully select breeding individuals based on certain traits. Among the possible approaches to selecting breeding individuals, we have the evaluation of the zoometric/linear appraisal traits of individuals. These traits refer to specific characteristics of the animal, such as height, weight, and body shape, that are indicative of their production potential. Similarly, bone quality and leg structure are crucial for the overall health and longevity of the animal (13).

To assess these traits accurately, breeders often rely on PBVs, which are calculated based on the animal’s genetic information and their phenotypic traits. PBVs allow breeders to identify animals with desirable genetic traits, even if those traits are not readily measurable in the animal (14).

Extensive studies have shown that there is a strong phenotypic and genetic relationship between morphological measurements and growth, reproduction, and milk production parameters. Morphometric traits provide explicit baseline information for characterizing goat and sheep breeds, which can be used in conventional animal breeding schemes, particularly in developing countries. Morphological traits can be incorporated into community-based animal breeding performance recording schemes, as they correspond to the functionality of animals for the purpose of production and partly confirm a possible but initial appraisal of the selection of animals’ reproductive and/or milk production capacity.

The testicular (male), pelvic (female), and udder morphometry have been the basis of application of morphological indices in reproductive and milk production performance assessment of type and function in goat and sheep production. Udder morphological characteristics values are significant indicators of the milking capacity of individual animals in dairy goat and sheep enterprises, and udder size is strongly and positively correlated with milk yield. The review recommends the incorporation of highly correlated linear type traits through the development of genotypic characterization in community-based breeding schemes. This will feed into genetic improvement strategies and productivity schemes. In most cases, the number of conformation traits recorded in conventional breeding schemes is relatively small because it might be expensive. Therefore, there is a need to focus on developing methods to measure morphological traits that are rapid and accurate at quantifying multiple conformation measurements while minimizing costs.

Our study moves from the investigation of the mere relationship between zoometry and milk production and composition to the relationship between breeding values for zoometric/linear appraisal breeding values and casein haplotypes. The casein haplotype structure varies greatly across breeds. However, its study is still scarce, particularly in species other than the cow. This translates into a patent lack of documents to compare with. Our results suggest that no differences in zoometric/linear appraisal related traits may be ascribed to the different haplotypic forms of the αS1, αS2, and κ Casein genes (Supplementary Table S2).

Particularly, some authors, such as Pizarro Inostroza et al. (10), reported that the expression of certain β casein haplotypes, together with specific haplotypes from the other casein loci, may indeed be linked to differential expressions of milk yields and composition and somatic cell counts. Certain sequences of αS1 casein and β casein loci were found to be associated with higher milk yields in Murciano-Granadina goats, with variations in fat, protein, dry matter, and lactose percentages. The haplotypic sequences GGGACCCC, GGAACCCC, and GGGATCTC had milk yields of 2.34, 2.45, and 2.45 kg, respectively. The combination of αS1 casein sequence GAGAAATCGAGAGAGCGA with β casein locus sequence GGGATCTC had the highest milk yield of 2.63 kg, but with lower fat, protein, and dry matter percentages. The combination of the αS1 casein sequence GAGGAATTAAAAGAGCAA with the β casein sequence GGGACCCC characterized an average milk yield of 3.73 kg, with lower fat, protein, and dry matter percentages and an increased lactose percentage of 4.88%. These sequences differed in the change of the alleles A → G, A → G, T → C, and T → C at SNPs 34, 35, 36, and 37, respectively (11). The presence of β casein haplotypic sequences GAGACCCC, GGAACCCC, GGAACCTC, GGAATCTC, GGGACCCC, GGGATCTC, and GGGGCCCC, linked to differential combinations of increased quantities of higher quality milk in terms of its composition, may also be connected to increased zoometric/linear appraisal predicted breeding values. To the best of our knowledge, no study has yet approached the relationship between casein haplotype and zoometric/linear appraisal traits from a genetic perspective. Our results suggest that haplotypic sequences within the β casein gene, such as GAGACCCC, GGAACCCC, GGAACCTC, GGAATCTC, GGGACCCC, GGGATCTC, and GGGGCCCC, which have been reported to be linked to differential combinations of increased quantities of higher quality milk in terms of its composition, are also linked to increased breeding values for zoometric/linear appraisal traits. An insufficient representativity of the animals presenting the GGAATCCC and GGAATTTT haplotypes was found, hence the lack of possibilities to determine the association between their presence and increased predicted breeding values for zoometric/linear appraisal traits. For those sequences for which no relevant associations with milk yield and component traits had been reported in the literature, such as GGAACCTT and GGGATCCC, maximum predicted values were low and even negative for important dairy-type-related traits. For certain haplotypic sequences, such as GGGACCTC, evaluation may be rather complex given they may participate in a rather conjoint effect together with the haplotypic sequences for other genes, such as αS1 and αS2 casein gene haplotypes. Indeed, it is the differential combinations that can appear that determine the wide range of milk yield and composition levels, from low to very high, as found by Pizarro Inostroza et al. (10).

The different haplotype combinations can be determined when the β casein locus is considered to exert a strongly favorable effect on milk yield and composition, in which the presence of the alleles A, G, T, and C is related to higher production and composition percentages. Contrastingly, G and T alleles may imply a reduction in somatic cell counts. However, this contrasts the finding by Baltrėnaitė et al. (65), who did not find statistically significant differences in milk performance across the different allelic combinations within the β casein locus. In this context, Chessa et al. (66) reported C may be the most frequent allele to appear within the β casein locus.

Pizarro Inostroza et al. (10) reported the effect of β casein haplotypes on milk yield and composition, rather than an isolated effect that should be considered in combination with the haplotypic sequences of other casein genes. In this regard, conjoined actions seem to be exerted that, in turn, not only modify the expression for milk performance and composition, as was also suggested by other authors (67), but also may condition dairy morphology type.

Consequently, the GGGATCCC haplotype may indeed be linked to reduced PBVs for several zoometric/linear appraisal traits, including those for rear insertion height, bone quality, anterior insertion, udder depth, rear legs side view, and rear legs rear view. This, in turn, means that goats carrying this haplotype may not be ideal for breeding if the goal is to improve these specific traits. However, it is important to note that the relationship between the GGGATCCC haplotype and these traits is not fully understood. Further research is needed to clarify the genetic basis of this association and to determine whether it is a causal relationship or simply a correlation.

By contrast, the GGAACCCC haplotype has been associated with increased PBVs for several zoometric/linear appraisal traits, including rear insertion height, bone quality, anterior insertion, udder depth, rear legs side view, and rear legs rear view. This makes goats carrying this haplotype highly desirable for breeding if the goal is to improve these traits.

Given the importance of the β casein gene in milk production and the potential impact of these haplotypes on zoometric/linear appraisal traits, the inclusion of genotype or haplotype for β casein in the stud catalogs of the Murciano-Granadina breed is recommended, alongside those of αS1 and κ casein, which are routinely tested for in merit bucks. By routinely testing for these haplotypes, breeders would be able to identify animals that have the potential to genetically transmit desirable traits to their offspring and avoid breeding animals that may have negative effects on certain traits. This can help maintain the overall quality and productivity of the Murciano-Granadina breed and ensure that it remains a valuable contributor to the dairy industry.

## 5. Conclusion

In conclusion, although the relationship between β casein haplotypes and zoometric/linear appraisal traits in Murciano-Granadina goats is not fully understood, inclusion of the genotype or haplotype for β casein in stud catalogs is highly recommended. This will enable breeders to identify animals that have the potential to genetically transmit desirable traits to their offspring and avoid breeding animals that may have negative effects on certain traits. Ultimately, this can help to maintain the overall quality and productivity of Murciano-Granadina goats.

## Data availability statement

The datasets presented in this study can be found in online repositories. The names of the repository/repositories and accession number(s) can be found in the article/Supplementary material.

## Ethics statement

Ethical review and approval was not required for the study on animals in accordance with the local legislation and institutional requirements.

## Author contributions

JF, FN, and AG: conceptualization and writing—original draft preparation. FN and JL: methodology, data curation, software, and visualization. MM and JD: validation, formal analysis, resources, and funding acquisition. JF, FN, AG, CP, and MP: investigation. JD: project administration. FN and AG: writing—review and editing. FN: supervision. All authors have read and agreed to the published version of the manuscript.

## Funding

The present research was developed under the context of a Ramón y Cajal Post-Doctoral fellowship financially supported by MCIN/AEI/10.13039/501100011033 and European Union Europea “NextGenerationEU”/PRTR” (Recovery, Transformation and ResiliencePlan—Funded by the European Union—NextGenerationEU).

## Conflict of interest

The authors declare that the research was conducted in the absence of any commercial or financial relationships that could be construed as a potential conflict of interest.

## Publisher’s note

All claims expressed in this article are solely those of the authors and do not necessarily represent those of their affiliated organizations, or those of the publisher, the editors and the reviewers. Any product that may be evaluated in this article, or claim that may be made by its manufacturer, is not guaranteed or endorsed by the publisher.
