# Supplementary material for: Discriminant canonical tool for inferring the effect of αS1, αS2, β, and κ casein haplotypes and haplogroups on zoometric/linear appraisal breeding values in Murciano-Granadina goats
Source: Front Vet Sci. 2023 Jul 7;10:1138528. doi: 10.3389/fvets.2023.1138528 (PMC10360128; doi:10.3389/fvets.2023.1138528)
Supplement: Supplementary file 1 [file Table_1.DOCX]

**Table S1.** Detailed description of the scales used and the translation process from zoometric traits to LAS scores in Murciano-Granadina goat and bucks.

| **Gender/Status** | **Major area** | **Linear trait** | **Zoometric Scale/ Categorical Scale** | **Zoometric Optimum Scoring** | **Reference/Middle point** | **LAS Extrapolation** | **LAS Optimum scoring** | **New Scale Proposal** **(Fernández Álvarez et al., 2022)** |
| --- | --- | --- | --- | --- | --- | --- | --- | --- |
| Primipara/Multipara does | Structure and capacity | Stature (Height to withers) | 62-78 cm | 72 cm (primipara) and 74 cm (multipara) | 5 (70 cm) | 1-9 points | 6 (primipara) and 7 (multipara) | 1-9 points |
|  |  | Chest Width | 15-23 cm | 20 cm (primipara) and 21 cm (multipara) | 5 (19 cm) | 1-9 points | 6 (primipara) and 7 (multipara) | 1-9 points |
|  |  | Body Depth | Shallow-Extremely deep | Intermediate | 5 (elbow end matches rib depth) | 1-9 points | 7 (primipara and multipara) | 1-8 points |
|  |  | Rump Width | 13-21 cm | 18 cm (primipara) and 19 (multipara) | 5 (17 cm) | 1-9 points | 6 (primipara) and 7 (multipara) | 1-7 points |
|  |  | Rump Angle | 55º-31º | 31º | 5 (43º) | 1-9 points | 9 | 1-7 points (Not relevant) (Fernández Álvarez et al., 2020) |
|  | Dairy structure | Angulosity | Angulous extremity-Rough extremity | Angulous extremity | 5 (Intermediate) | 1-9 points | 9 | 1-10 points |
|  |  | Bone Quality | Round and rough bones-flat and neat bones | Flat and neat bones | 5 (Intermediate) | 1-9 points | 9 | 1-5 points |
|  | Mammary system | Anterior insertion | Weak-Strong | 120º | 5 (90º) | 1-9 points | 9 | 1-5 points |
|  |  | Rear Insertion Height | 11-3 cm | 3 cm | 5 (7 cm) | 1-9 points | 9 | 1-5 points |
|  |  | Median Suspensor Ligament | 1-9 cm | 5 cm | 5 (5 cm) | 1-9 points | 5 | 1-6 points |
|  |  | Udder width | 3-11 cm | 11 cm | 5 (7 cm) | 1-9 points | 9 | 1-5 points |
|  |  | Udder Depth | -10-10 cm | -5 cm (5 cm over hock level) and 0 cm (udder bottom at hock level) | 5 (0 cm/at hock level) | 1-9 points | 3 (primipara) and 5 (multipara) | 1-9 points |
|  |  | Nipple placement | 90º-0º | 0º | 5 (45º) | 1-9 points | 9 | 1-6 points |
|  |  | Nipple Diameter | 0.5º to 4.5º | 2 cm | 5 (2.5 cm) | 1-9 points | 4 | 1-9 points |
|  | Legs aplomb | Rear Legs Rear View | Very close-Parallel and separated | Parallel and separated | 5 (slightly close) | 1-9 points | 9 | 1-7 points |
|  |  | Rear Legs Side View | Straight-Very curved | Desirable curvature. A short distance from an imaginary line to anterior curvature of hock | 5 (desirable curvature) | 1-9 points | 5 | 1-7 points |
|  |  | Mobility | Very bad mobility due to skeleton structure-long and strong, straight and uniform stride | Good mobility. Easy and harmonic movement | 5 (moderate mobility) | 1-9 points | 9 | 1-5 points |
| Bucks | Structure and capacity | Stature (Height to withers) | 68-92 cm | 83 cm (young) and 86 cm (adult) | 5 (80 cm) | 1-9 points | 6 (bucklings) and 7 (bucks) | 1-10 points |
|  |  | Chest Width | 15-31 cm | 25 cm (young) and 27 cm (adult) | 5 (23 cm) | 1-9 points | 6 (bucklings) and 7 (bucks) | 1-11 points |
|  |  | Body Depth^a^ | Shallow-Extremely deep | Intermediate | 5 (elbow end matches rib depth) | 1-9 points | 7 (bucklings and bucks) | 1-7 points |
|  |  | Rump Width | 14-22 cm | 19 cm (young) and 20 cm (adult) | 5 (18 cm) | 1-9 points | 6 (bucklings) and 7 (bucks) | 1-5 points |
|  |  | Rump Angle | 55-31º | 31º | 5 (43º) | 1-9 points | 9 | 1-6 points |
|  | Dairy structure | Angulosity^a^ | Angulous extremity-Rough extremity | Angulous extremity | 5 (Intermediate) | 1-9 points | 9 | 1-9 points |
|  |  | Bone Quality^a^ | Round and rough bones-flat and neat bones | Flat and neat bones | 5 (Intermediate) | 1-9 points | 9 | 1-5 points |
|  | Legs aplomb | Rear Legs Rear View^a^ | Very close-Parallel and separated | Parallel and separated | 5 (slightly close) | 1-9 points | 9 | 1-6 points |
|  |  | Rear Legs Side View^a^ | Straight-Very curved | Desirable curvature. Short distance from an imaginary line to anterior curvature of hock | 5 (desirable curvature) | 1-9 points | 5 | 1-7 points |
|  |  | Mobility^a^ | Very bad mobility due to skeleton structure-long and strong, straight and uniform stride | Good mobility. Easy and harmonic movement | 5 (moderate mobility) | 1-9 points | 9 | 1-5 points |
| ^a^Same criteria for bucks and does. | | | | | | | |  |
